# Ruthenium nanoparticles confined in SBA-15 as highly efficient catalyst for hydrolytic dehydrogenation of ammonia borane and hydrazine borane

**DOI:** 10.1038/srep15186

**Published:** 2015-10-16

**Authors:** Qilu Yao, Zhang-Hui Lu, Kangkang Yang, Xiangshu Chen, Meihua Zhu

**Affiliations:** 1 Jiangxi Inorganic Membrane Materials Engineering Research Centre, College of Chemistry and Chemical Engineering, Jiangxi Normal University, Nanchang 330022, China

## Abstract

Ultrafine ruthenium nanoparticles (NPs) within the mesopores of the SBA-15 have been successfully prepared by using a “double solvents” method, in which *n*-hexane is used as a hydrophobic solvent and RuCl_3_ aqueous solution is used as a hydrophilic solvent. After the impregnation and reduction processes, the samples were characterized by XRD, TEM, EDX, XPS, N_2_ adsorption-desorption, and ICP techniques. The TEM images show that small sized Ru NPs with an average size of 3.0 ± 0.8 nm are uniformly dispersed in the mesopores of SBA-15. The as-synthesized Ru@SBA-15 nanocomposites (NCs) display exceptional catalytic activity for hydrogen generation by the hydrolysis of ammonia borane (NH_3_BH_3_, AB) and hydrazine borane (N_2_H_4_BH_3_, HB) at room temperature with the turnover frequency (TOF) value of 316 and 706 mol H_2_ (mol Ru min)^−1^, respectively, relatively high values reported so far for the same reaction. The activation energies (*E*_a_) for the hydrolysis of AB and HB catalyzed by Ru@SBA-15 NCs are measured to be 34.8 ± 2 and 41.3 ± 2 kJ mol^−1^, respectively. Moreover, Ru@SBA-15 NCs also show satisfied durable stability for the hydrolytic dehydrogenation of AB and HB, respectively.

Hydrogen, a clean and renewable fuel, is considered as a potential energy carrier for future energy infrastructure^1^. Storing hydrogen safely and efficiently still remains a great challenge. Currently, many types of hydrogen storage materials have been studied and found to be promising for hydrogen storage^2^,^3^,^4^. Among these hydrogen storage materials, ammonia borane (NH_3_BH_3_, AB) was considered as one of the leading candidates for on-board hydrogen application owing to its high hydrogen content (19.6 wt%), low molecular weight, non-toxicity, and stable in neutral aqueous solution^5^,^6^. The closely compound hydrazine borane (N_2_H_4_BH_3_, HB) with the hydrogen capacity of 15.4 wt% has become another potential B-N based hydrogen storage material^7^,^8^,^9^. Hydrogen stored in AB and HB can be released through different ways, such as thermal dehydrogenation in solid state^10^,^11^ and solvolysis in solution (hydrolysis^12^,^13^,^14^,^15^,^16^ and methanolysis^17^,^18^). However, thermal dehydrogenation process needs high temperature and the reaction is relatively difficult to control. In contrast, with appropriate catalyst, hydrolysis of AB (eqn 1) and HB (eqn 2) can be released 3.0 equivalents of hydrogen at room temperature, which seems to be the most convenient route for portable hydrogen storage applications^5^,^19^,^20^,^21^.


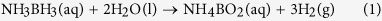



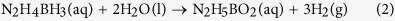


So far a lot of metal nanocatalysts have been tested for hydrogen generation from the hydrolysis of AB and HB^22^,^23^,^24^,^25^,^26^,^27^,^28^,^29^,^30^,^31^,^32^,^33^, among which platinum group metals including Pt, Rh, and Ru based catalysts display a remarkable performance in hydrogen production as compared to other catalysts even at a very low metal concentration^14^,^19^,^31^,^32^,^33^. Although, platinum group metals have limited resources and high price tags, the use of them as catalysts for the hydrolysis of AB and HB still have great significance. With the development of nanoscience and technology, nanoscaled metal particles have attracted extensive attention in the past decades owing to their distinctive properties as compared to their bulk phase^34^,^35^. However, metal NPs easily aggregate due to their high surface energy, thus resulting in reducing their surface area and accessible active sites available for catalytic reactions. In order to improve the stability of metal NPs, several routes have been adopted, mainly by capping of metal NPs with surfactants during reaction or by using support with high specific surface area^36^,^37^,^38^,^39^. Compared to the conventional support materials such as zeolites^40^, carbon black^41^, Al_2_O_3_^42^, SiO_2_^37^, TiO_2_^43^, MCM-41^44^, SBA-15 has a short history, which was firstly synthesized and reported by Zhao *et al.*^45^. However, it attracted rapid attention in materials science and catalysis, and has been emerged as a potential support for growing and anchoring metal NPs due to its ordered structure, narrow and controllable pore size and large specific surface area^46^,^47^,^48^,^49^,^50^,^51^. Given the similarity to zeolites, immobilizing metal NPs to SBA-15 could obtain well dispersed catalysts. More important, the encapsulation of metal NPs into the mesopores of SBA-15 could effectively prevent the coalescence of metal NPs due to the confinement effects in those materials and at the same time provide channels for the reactants to reach the surface of metal NPs, thus allowing catalytic reaction to occur.

In this work, ultrafine Ru NPs with an average size of 3.0 ± 0.8 nm were confined in the mesopores of SBA-15 by using a double solvents method. The obtained Ru@SBA-15 NCs exhibited excellent catalytic activity in the hydrolysis of AB and HB at room temperature with total turnover frequencies (TOF) of 316 and 706 mol H_2_ (mol Ru min)^−1^, respectively. Moreover, Ru@SBA-15 NCs also show good stability for the hydrolytic dehydrogenation of AB and HB under mild condition. Additionally, the kinetics of hydrolysis of AB and HB by Ru@SBA-15 NCs were studied under different catalyst concentrations, substrate concentrations, and reaction temperatures.

## Results

**Figure 1:**
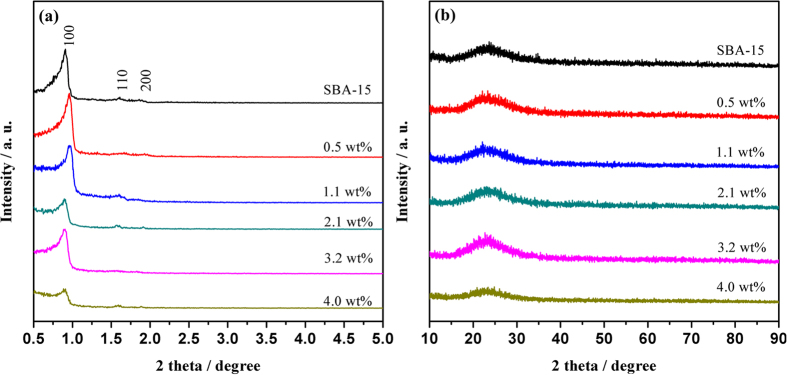
(**a**) Small-angle and (**b**) wide-angle XRD patterns for the synthesized Ru@SBA-15 NCs with different Ru loadings.

Ultrafine Ru NPs within the pores of the mesoporous SBA-15 were prepared via a double solvents technique, in which *n*-hexane is used as a hydrophobic solvent to suspend SBA-15 and RuCl_3_ aqueous solution is used as a hydrophilic solvent to fill mesopores. After the impregnation and reduction processes, the obtained Ru@SBA-15 NCs were isolated from the reaction solution by centrifugation and characterized by XRD, SEM, TEM, EDX, and XPS techniques. Figure 1 present the small-angle and wide-angle XRD patterns, respectively, for the as-synthesized Ru@SBA-15 NCs with different Ru loadings. As shown in Fig. 1a, both of pure SBA-15 and Ru@SBA-15 NCs with different Ru loadings show three peaks at 2θ = 0.90°, 1.59°, and 1.89° which indexed to the (100), (110), and (200) of the hexagonal mesoporous structure, indicating that the framework of SBA-15 was maintained after confining Ru NPs. While, the (100) diffraction peaks of 0.5 wt% and 1.1 wt% of Ru@SBA-15 NCs samples in Fig. 1(a) shifted to higher angle probably because of a large incorporation of Ru NPs within the pores of SBA-15. As shown in Fig. 1b, the strong and broad peaks at 2*θ* = 15°–35° can be assigned to the amorphous structure of the SBA-15. As for the Ru@SBA-15 NCs with different Ru loadings, there is only amorphous diffraction peaks, with no Ru NPs characteristic diffractions being detected in the wide-angle PXRD patterns (Fig. 1b), probably due to the fact that the Ru loading of Ru@SBA-15 NCs is too low. For Ru@SBA-15 NCs with a higher loading (20 wt%), the diffraction peak attributed to Ru (JCPDS no. 06-0663) were observed (Fig. S2).

**Figure 2:**
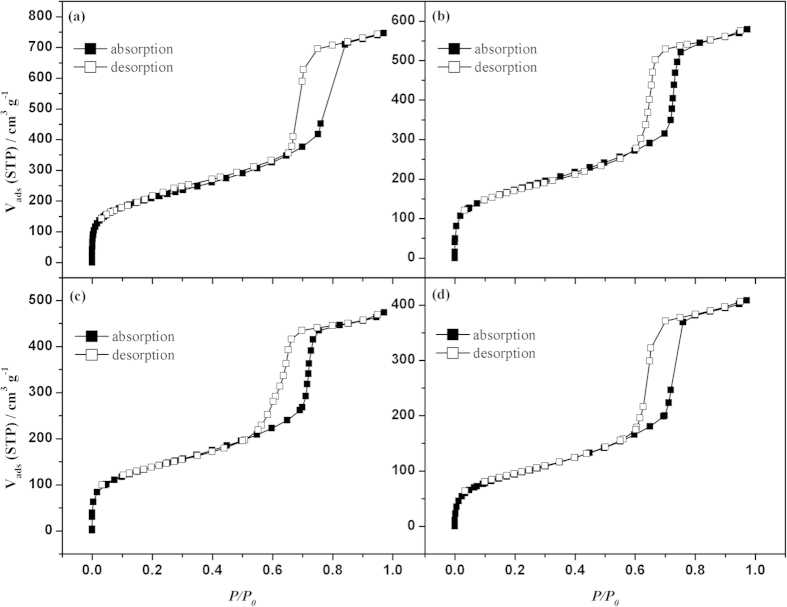
Nitrogen adsorption-desorption isotherms of the (**a**) SBA-15, (**b**) 0.5 wt% Ru@SBA-15, (**c**) 2.1 wt% Ru@SBA-15, and (**d**) 4.0 wt% Ru@SBA-15 NCs.

Figure 2 shows the nitrogen adsorption-desorption isotherms for SBA-15 and Ru@SBA-15 NCs with different Ru loadings (0.5 wt%, 2.1 wt%, and 4.0 wt%). As shown in Fig. 2, all the samples present type IV isotherms with H1 hysteresis loops in the relative pressure (P/P_0_) between 0.6 and 0.8, characteristic of highly ordered mesoporous materials. This reveals that the ordered mesoporous structure has been maintained after the loading of Ru NPs. The corresponding pore size distributions calculated by Barrett-Joyner-Halenda (BJH) method are around 7 nm (Fig. S3), which is consist with the previous studies^45^. The BET surface areas of SBA-15, 0.5 wt% Ru@SBA-15, 2.1 wt% Ru@SBA-15 and 4.0 wt% Ru@SBA-15 were calculated to be 673, 541, 438 and 319 m^2^ g^−1^, respectively. The decreases of surface areas could be attributed to the incorporation of Ru NPs into the pores of SBA-15 and/or the block by the Ru NPs.

**Figure 3:**
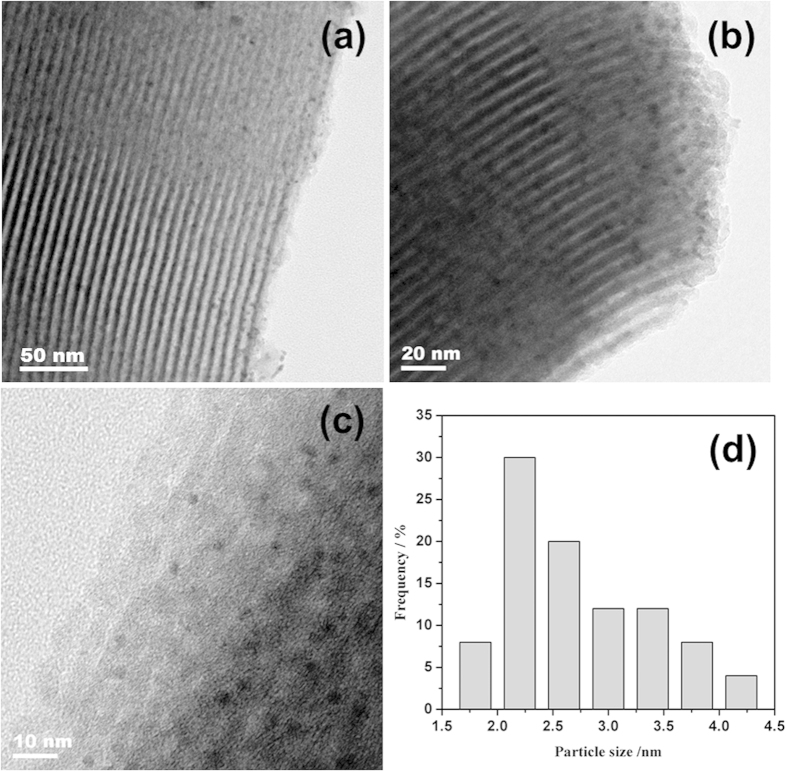
(**a**–**c**) TEM images and (**d**) particle distribution of Ru@SBA-15 NCs with 2.1 wt% Ru loading.

**Figure 4:**
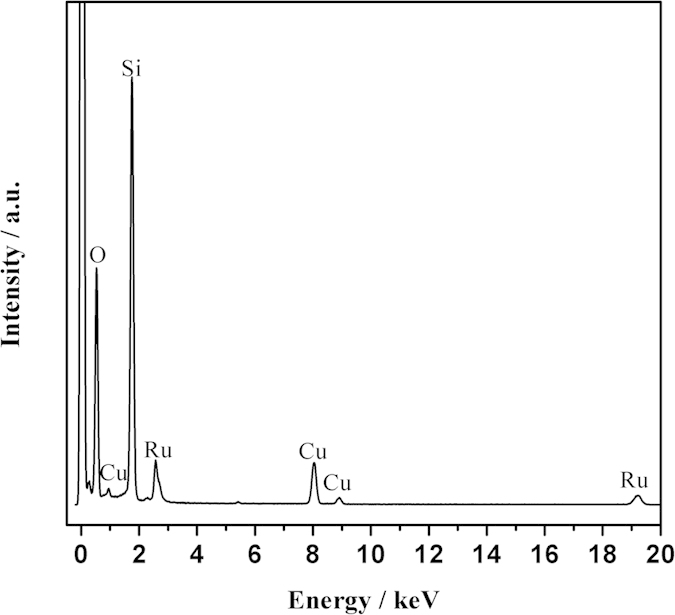
The corresponding EDX spectrum of the Ru@SBA-15 NCs with 2.1 wt% Ru loading. The Cu signal originates from Cu grid.

The size and morphology of Ru@SBA-15 NCs were observed by transmission electron microscopy (TEM). As shown in Fig. 3, the Ru@SBA-15 NCs (2.1 wt%) display a well-ordered mesoporous channel structure, which is good agreement with the small angle XRD patterns (Fig. 1a) and nitrogen adsorption-desorption measurements (Fig. 2). TEM images of Ru@SBA-15 NCs (2.1 wt%) show that Ru NPs are highly dispersed in the mesopores of SBA-15 (Fig. 3a–c). TEM images of Ru@SBA-15 NCs with other Ru loadings (0.5 wt%, 1.1 wt%, and 4.0 wt%) were given in Fig. S4. These TEM images show that Ru@SBA-15 NCs with a high Ru loading (4.0 wt%) also show no large particle aggregation. As shown in Fig. 3d and Fig. S4, the sizes of Ru NPs are about 2.0 ± 0.6 nm, 2.2 ± 0.6 nm, 3.0 ± 0.8 nm, and 3.7 ± 0.7 nm for the 0.5 wt.%, 1.1 wt.%, 2.1 wt.%, and 4.0 wt.% Ru loading of Ru@SBA-15 NCs, respectively, which are small enough to encapsulate into the mespores of SBA-15 (*ca.* 7.0 nm). Thus, the reactant molecules can readily access the Ru NPs through the mesopores and the product molecules can be easily exit through these pores. Ru is the only element detected by using EDX analysis, in addition to the SBA-15 framework elements (Si, O) and Cu from the TEM grid (Fig. 4).

**Figure 5:**
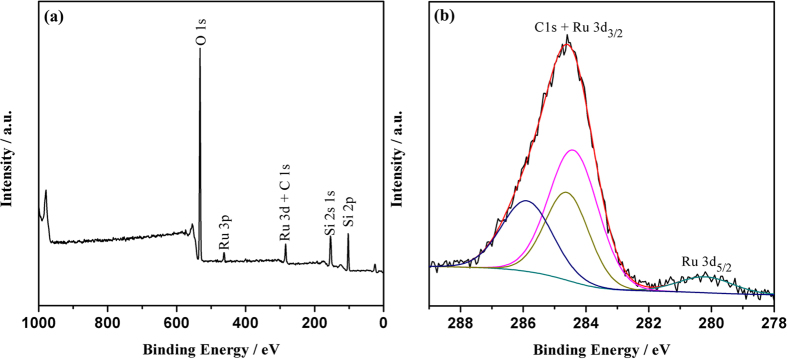
X-ray photoelectron (XPS) spectrum of (**a**) the survey scan and (**b**) Ru 3d peaks of the Ru@SBA-15 NCs with 2.1 wt% Ru loading.

To better understand the composition of Ru@SBA-15 NCs, we further carried out XPS analysis. Figure 5a shows the XPS survey scan spectrum of Ru@SBA-15 NCs with a Ru loading of 2.1 wt.%. The survey scan spectrum reveals that Ru is the only element detected except the SBA-15 framework elements (Si, O). This is well consistent with the EDX result. The high resolution Ru 3d XPS spectrum shows two prominent peaks at 284.6 and 280.2 eV, which can be readily assigned to 3d_3/2_ and 3d_5/2_ of Ru(0), respectively (Fig. 5b). It is notable that the overlap of the Ru 3d and C 1s peaks around at 285 eV makes it difficult to confirm the presence of Ru. While in the XPS survey scan (Fig. 5a), the Ru 3p peak around at 463 eV can be observed to identify the existence of Ru. In addition, a small amount of Ru oxides was observed at 285.9 eV in the XPS spectrum of Ru@SBA-15, which is due to the surface oxidation of Ru through a strong interaction of Ru with SBA-15 before the XPS measurement as evidenced by the H_2_-TPR profile (Fig. S5).

## Discussion

**Figure 6:**
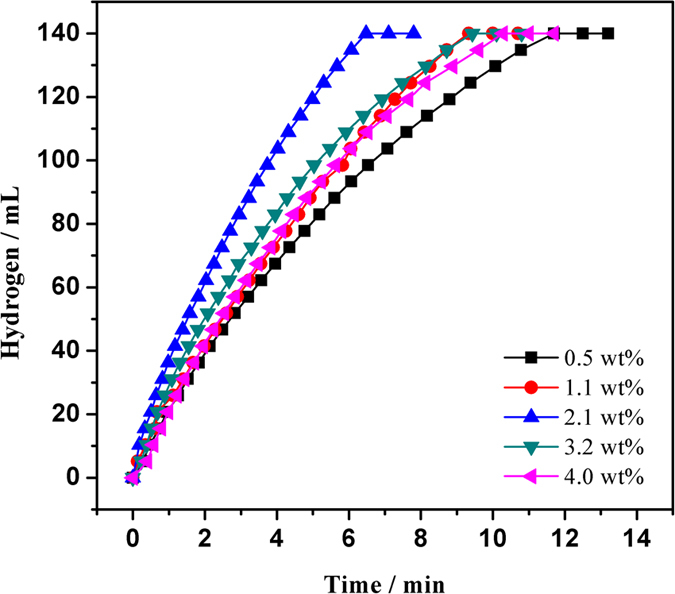
Hydrogen generation from the hydrolysis of AB (200 mM, 10 mL) catalyzed by Ru@SBA-15 NCs with different Ru loadings at 298 K (Ru/AB = 0.002).

Figure 6 shows the catalytic activities of Ru@SBA-15 NCs with different Ru loadings in the range of 0.5–4.0 wt% for H_2_ generation from the hydrolysis of AB at 298 K. The hydrogen generation rate significantly relies on the loading of Ru. As shown in the Fig. 6, a stoichiometric amount of hydrogen (140 mL) is evolved in 11.70, 9.33, 6.48, 9.43, 10.28 min in the presence of the Ru@SBA-15 NCs with Ru loading of 0.5, 1.1, 2.1, 3.2 and 4.0 wt%, respectively. Obviously, Ru@SBA-15 NCs with Ru loading of 2.1 wt.% exhibit the highest catalytic activity with a TOF value as high as 316 mol H_2_ (mol Ru min)^−1^, relatively high value for the Ru-based catalysts tested for the same reaction (Table S1)^34^,^35^,^37^,^38^,^40^,^41^,^42^,^43^,^52^,^53^,^54^,^55^,^56^. However, no hydrogen gas generation was observed for the SBA-15 support, suggesting that SBA-15 is inactive for the hydrolysis of AB (Fig. S7). The catalyst of Ru@SBA-15 (2.1 wt%) also show a higher catalytic activity for the hydrolysis of AB than that of Ru/SBA-15 NCs (2.1 wt%), Ru/SiO_2_ NCs (2.1 wt%), and free Ru NPs (Fig. S7). The excellent catalytic activity of Ru@SBA-15 NCs could be attributed to the good dispersion of Ru NPs and the strong interaction between Ru and SBA-15 support (Fig. S5). More important, the reactant molecules can readily access the Ru NPs through the mesopores and the product molecules can be easily exit through these pores. Thus, Ru@SBA-15 catalyst with Ru loading of 2.1 wt% was selected to be used in all of the experiments for the further investigation.

**Figure 7:**
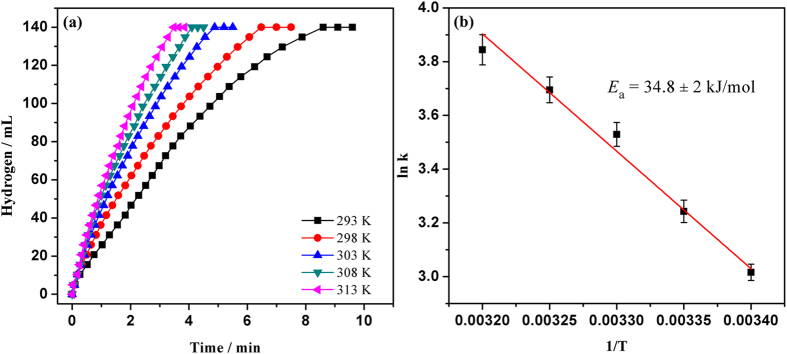
(**a**) Hydrogen generation from the hydrolysis of AB (200 mM, 10 mL) catalyzed by Ru@SBA-15 NCs at 293–313 K (Ru/AB = 0.002). (**b**) Arrhenius plot: ln k versus 1/T.

To study the kinetics of the hydrolysis of AB catalyzed by Ru@SBA-15 NCs, a series of experiments were carried out. Figure S8a shows the plots of hydrogen generated versus reaction time during the hydrolysis of AB at different catalyst concentrations. The hydrogen generation rates for each Ru concentration were determined from the linear portion of each plot. Figure S8b displays the logarithmic plot of the hydrogen generation rate vs. Ru concentration. The slop of the obtained line is found to be 0.96, which is closed to 1, indicating that the catalytic hydrolysis of AB is a first-order reaction with respect to the catalyst concentration. The effect of AB concentration on the hydrogen generation rate was examined by carrying out at various initial concentration of AB in the range of 100–500 mM, as shown in Fig. S9a. The straight line with a slop of 0.15 in Fig. S9b implies that the hydrolysis reaction is zero order with respect to the AB concentration. To determine the effect of H_2_O, different volumes of H_2_O (5–50 mL) were employed for the same catalytic reaction (Fig. S10a). As shown in Fig. S10b, the slop of the line is determined to be 0.05, indicating the catalytic hydrolysis of AB can be described as a zero order reaction with respect to the volume of H_2_O. In addition, it was found that the hydrogen generation rate generally increases with the increase of the reaction temperature (Fig. 7). The activation energy (*E*_a_) for the hydrolysis of AB is determined by the Arrhenius plot to be 34.8 ± 2 kJ mol^−1^, being lower than most of the reported *E*_a_ values of for the same reaction by using many different catalysts (Table S1)^34^,^35^,^37^,^38^,^40^,^41^,^42^,^43^,^52^,^53^,^54^,^55^,^56^, indicating that the Ru@SBA-15 NCs exhibit superior catalytic performance.

**Figure 8:**
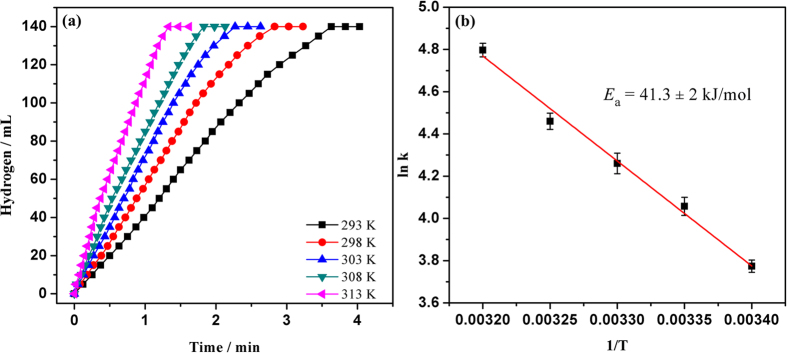
(**a**) Hydrogen generation from the hydrolysis of HB (200 mM, 10 mL) catalyzed by Ru@SBA-15 NCs at 293–313 K (Ru/HB = 0.002). (**b**) Arrhenius plot: ln k versus 1/T.

Ru@SBA-15 NCs with loading of 2.1 wt% was also applied as a catalyst for the hydrogen generation from the hydrolysis of HB at room temperature, and a series of kinetics experiments were performed. Fig. S11a shows the plots of hydrogen generation from the hydrolysis of 200 mM HB solution in the presence of Ru@SBA-15 NCs with the different Ru concentrations at 298 K. The slope of the line given in the Fig. S11b is 1.05, suggesting that the hydrolysis of HB catalyzed by Ru@SBA-15 NCs is first-order reaction with respect to the catalyst concentration. The effect of HB concentration in the range of 100–500 mM on the hydrogen generation rate was studied at 298 K (Fig. S12a). The line slope of the plot of hydrogen evolution rate versus HB concentration on a logarithmic scale is 0.02 (Fig. S12b), revealing that the hydrolysis reaction is zero order with respect to the HB concentration. In addition, the effect of H_2_O volume on the catalytic performance was also investigated (Fig. S13). The result shows that the catalytic hydrolysis of HB is a zero order reaction with respect to the volume of H_2_O. From the Arrhenius plot of rate constant *k* over Ru@SBA-15 obtained in the range of 293–313 K (Fig. 8), the activation energy *E*_a_ for the hydrolysis of HB was calculated to be 41.3 ± 2 kJ mol^−1^, which is the lowest value ever reported for the catalytic hydrolysis of HB (Table S2)^14^−^16^,^30^. The TOF value for hydrogen generation from the hydrolysis of HB in the presence of Ru@SBA-15 NCs was calculated to be 706 mol H_2_ (mol Ru min)^−1^, which is the highest value among the catalysts ever reported for the hydrolysis of HB^14^,^15^,^16^,^30^. However, Ru@SBA-15 NCs can only release the hydrogen of HB by the hydrolysis of -BH_3_ group of HB. Recent researches have shown that the complete hydrogen generation from HB (5 equivalents of H_2_) can be achieved via both the hydrolysis of -BH_3_ group and the decomposition of the -N_2_H_4_ group by using Ni-M (M = Pt, Ru, Rh) nanocatalysts, which further enhances the importance of the use of HB as the chemical hydrogen storage^7^,^8^,^9^,^57^,^58^,^59^,^60^, However, the catalytic activity for the high-extent dehydrogenation of HB is relatively low in the presence of these nanocatalysts, and the highest TOF value reported so far is ~11 mol H_2_ (mol metal min)^−1^ by using Ni_0.6_Pt_0.4_/MSC-30 as the catalysts^57^. Our preliminary results show that the Ru-Ni@SBA-15 catalytic system displays a high activity for the complete hydrogen generation from HB and this research is still in process.

**Figure 9:**
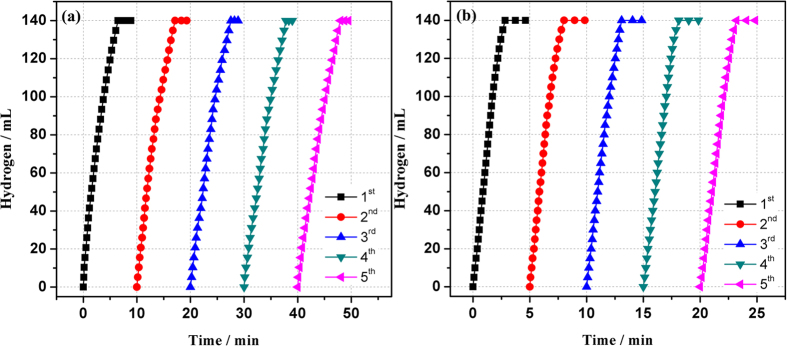
Durability test for the hydrogen generation from aqueous (**a**) AB and (**b**) HB solution (200 mM, 10 mL) in the presence of Ru@SBA-15 NCs at 298 K (Ru/AB = 0.002).

The durability of the catalyst is an important factor in the practical application. Hence, the durability of the Ru@SBA-15 NCs is examined by adding the same amount of AB or HB (2 mmol) into the reaction flask after the completion of the previous run. As shown in Fig. 9, the catalytic activity of Ru@SBA-15 NCs show no obvious decrease after five runs for the hydrolytic dehydrogenation of AB and HB. After the catalytic reaction, the morphology of Ru@SBA-15 NCs show no significant change and Ru NPs are still well dispersed in the pores of SBA -15 (Fig. S14), which is vital for maintaining the high catalytic activity of nanocatalysts. The above results clearly indicate that the ultrafine Ru NPs within the mesopores of SBA-15 can effectively prevent the coalescence of Ru NPs and exhibit excellent catalytic activity for the hydrolysis of AB and HB.

In summary, we have developed a simple double solvents method for the fabrication of Ru@SAB-15 NCs, in which the small sized Ru NPs are highly dispersed in the mesopores of SBA-15. The as-synthesized Ru@SBA-15 NCs exhibit excellent catalytic performance for the hydrolysis of HB and AB with the TOF values of 316 and 706 mol H_2_ (mol Ru min)^−1^, respectively. In addition, the activation energies for the hydrolysis of AB and HB catalyzed by Ru@SBA-15 NCs are found to be 34.8 ± 2 and 41.3 ± 2 kJ mol^−1^, respectively. Furthermore, the as-synthesized Ru@SBA-15 NCs possess excellent cycle stability for the hydrolytic dehydrogenation of AB and HB. The highly efficient catalysts, easily prepared via a double solvents method, represent a promising step toward the practical applications of mesoporous SBA-15 as effective matrices to confine metal NPs in the catalytic hydrolysis reaction system.

## Methods section

### Materials

Ammonia borane (NH_3_BH_3_, AB, Aldrich, 90%), tetraethoxysilane (TEOS, Aldrich, 98%), poly(ethylene glycol)-block-poly(propylene glycol)-block-poly(ethylene glycol) (P123, Aldrich), hydrochloric acid (Nanchang Chemical Works, 65 wt%), sodium borohydride (NaBH_4_, Aldrich, 99%), ruthenium(III) chloride hydrate (RuCl_3_·xH_2_O, Aladdin Industrial Inc, 38~42 wt% Ru basis), commercial SiO_2_ (specific surface area = 200 m^2^ g^−1^), Degussa, 99.8%), *n*-hexane (C_6_H_14_, Tianjin Fuchen Chemical Reagent, >99.5%), *n*-pentane, and hydrazine hemisulfate ((N_2_H_4_)_2_∙H_2_SO_4_) were used without further purification. Ultrapure water with the specific resistance of 18.3 MΩ·cm was obtained by reversed osmosis followed by ion exchange and filtration.

### Characterization

Powder X-ray diffraction (XRD) patterns were performed at room temperature using an X-ray diffractometer (Rigaku RINT-2000) with Cu Kα radiation (40 kV, 40 mA). Transmission electron microscope (TEM, JEM-2010) equipped with an energy-dispersive X-ray detector (EDX) was applied for the detailed microstructure and composition of the synthesized samples. The TEM samples were dispersed in ethanol by sonication, and one or two droplets of the nanoparticle suspensions were dropped onto a carbon-coated copper grid and dried in air. X-ray photoelectron spectroscopy (XPS) was carried out on an ESCALABMKLL X-ray photoelectron spectrometer with Al Kα radiation. The surface area, pore diameter, and pore volume of the samples were derived from the nitrogen adsorption-desorption isotherms at 77 K using automatic volumetric adsorption equipment (Belsorp mini II). Ru contents of Ru@SBA-15 NCs samples were determined by an inductively coupled plasma-atomic emission spectroscopy (ICP-AES) after each sample was completely dissolved in the mixture of HNO_3_/HCl (1/3 ratio). Hydrogen temperature programmed reduction (H_2_-TPR) experiments were carried out on a Micromeritics AutoChem II 2920 automated catalyst characterization system. For H_2_-TPR measurement, 50 mg of sample was weighted and heated (10 °C min^−1^) from room temperature to 600 °C in a flow of 10% H_2_/Ar mixture. The metal dispersion of Ru (D_Ru_) on the SBA-15 was determined by hydrogen temperature programmed desorption (H_2_-TPD), using the same instrument as above.

### Synthesis of SBA-15

SBA-15 was synthesized following the literature procedure^45^. Briefly, 4.0 g P123 template dissolved in 94 mL water, followed by addition of 20 mL HCl. Under stirring, 8.8 g TEOS was then injected within 30 min and the mixture was stirred continuously for 24 h at 40 °C. Then, the gel mixture was transferred to an autoclave and hydrothermally treated at 100 °C for 24 h. The as-prepared sample was filtered, washed with deionized water until pH of the filtrate reached the value 7, and dried at 60 °C overnight. The organic template was removed by calcination at 550 °C in air for 6 h, giving SBA-15 mesoporous material as a white powder.

### Synthesis of Ru@SBA-15 NCs with different Ru loadings

Ru@SBA-15 NCs with different Ru loadings were carried out via a double solvents method^19^,^51^. Briefly, 200 mg SBA-15 was first dried at 140 °C for 4 h under vacuum. Then 40 mL *n*-hexane as the hydrophobic solvent was added to the dry SBA-15 with sonication for 15 min to get the well-dispersed SBA-15 suspension. After stirring for 2 h, 0.2 mL of aqueous RuCl_3_·xH_2_O solution with desired concentrations as the hydrophilic solvent was added dropwise with continuous stirring for 2 h. After filtration, the brown powder was dried at room temperature in air. These as-synthesized samples were further dried at 423 K for 12 h under vacuum. The obtained samples were reduced with fresh NaBH_4_ (0.6 M, 5 mL) aqueous solution, centrifuged, washed, and then used for the catalytic reactions. The synthesized Ru@SBA-15 NCs with different Ru loadings (0.5, 1.1, 2.1, 3.2, 4.0 wt%) were determined by ICP-AES.

### Catalytic activity of Ru@SBA-15 NCs with different Ru loadings in hydrolysis of AB

Typically, a mixture of AB (2 mmol) and Ru@SBA-15 NCs with different Ru loadings were placed in a 50 mL two-necked round-bottomed flask. One neck of the flask was connected to a gas burette to measure the volume of hydrogen gas. The molar ratios of Ru/AB for all the hydrolysis reactions were kept as a constant of 0.002. The reaction started when 10 mL distilled water was injected into the flask with vigorous stirring. The volume of gas evolution was measured by recording the displacement of water in the gas burette. The reaction was ceased when there was no more gas evolved. The catalytic reactions were carried out at 298 K under ambient atmosphere. Ru@SBA-15 NCs with a Ru loading of 2.1 wt% exhibits the highest catalytic activity among all the synthesized catalysts with different metal loading. Thus, Ru@SBA-15 NCs catalyst with Ru loading of 2.1 wt% was selected to be used in all of the experiments for the further investigation.

### Kinetics study for hydrolysis of AB or HB catalyzed by Ru@SBA-15 NCs

The kinetics study for AB or HB hydrolysis reaction was tested on the Ru@SBA-15 NCs (Ru loading: 2.1 wt%) by varying catalyst concentrations, substrate concentrations, H_2_O volumes, and temperatures. In the first set of experiments, the concentration of AB or HB was kept constant at 200 mM and Ru concentration was varied in the range of 0.1–1.2 mM at 298 K. In the second set of experiments, Ru concentration was held constant at 0.4 mM while the AB or HB concentration was varied in the range of 100–500 mM at 298 K. In the third set of experiments, the molar content of AB or HB was kept constant at 2 mmol and the molar content of Ru was held constant at 0.004 mmol, while the volume of H_2_O was changed in the range of 5–50 mL at 298 K. Finally, in order to get the activity energy, the catalytic hydrolysis of AB or HB was tested at constant AB or HB concentration (200 mM, 10 mL) and Ru concentration (0.4 mM) at various temperatures in the range of 293–313 K.

### Durability for hydrolytic dehydrogenation of AB and HB

For testing the durability of the Ru@SBA-15 NCs, another equivalent of AB or HB (2 mmol) was subsequently added to the reaction system after the completion of the first run. Such test cycles of the catalyst for the hydrogen generation from the hydrolysis of AB or HB were carried out for five runs under ambient atmosphere at room temperature.

## Additional Information

**How to cite this article**: Yao, Q. *et al.* Ruthenium nanoparticles confined in SBA-15 as highly efficient catalyst for hydrolytic dehydrogenation of ammonia borane and hydrazine borane. *Sci. Rep.* **5**, 15186; doi: 10.1038/srep15186 (2015).

## Supplementary Material

Supplementary Information
