# Supplementary material for: Ruthenium nanoparticles confined in SBA-15 as highly efficient catalyst for hydrolytic dehydrogenation of ammonia borane and hydrazine borane
Source: Sci Rep. 2015 Oct 16;5:15186. doi: 10.1038/srep15186 (PMC4607937; doi:10.1038/srep15186)
Supplement: Supplementary Information [file srep15186-s1.doc]

**Supporting Information**

**Ruthenium nanoparticles confined in SBA-15 as highly efficient catalyst for hydrolytic dehydrogenation of ammonia borane and hydrazine borane**

*Qilu Yao, Zhang-Hui Lu,* Kangkang Yang, Xiangshu Chen,* Meihua Zhu*

Jiangxi Inorganic Membrane Materials Engineering Research Centre, College of Chemistry and Chemical Engineering, Jiangxi Normal University, Nanchang 330022, China

*E-mail: luzh@jxnu.edu.cn (Z.-H. Lu), cxs66cn@jxnu.edu.cn (X.S. Chen).

**Synthesis of Ru NPs supported on SBA-15 (Ru/SBA-15).** The Ru/SBA-15 in this study was prepared by the conventional impregnation and subsequent NaBH_4_ reduction. Briefly, 200 mg of SBA-15 is firstly dispersed in 5 mL of H_2_O with sonication for 30 min to get the well suspension. And then, 11.30 mg of RuCl_3_·xH_2_O was added to the above suspension. After vigorous stirring for 48 h, the fresh NaBH_4_ aqueous solution (0.6 M, 5 mL) was added into the above mixture with vigorous stirring. After about 20 min, the product was centrifuged, washed, dried, and then used for the catalytic reactions.

**Synthesis of Ru NPs supported on commercial SiO_2_ (Ru/SiO_2_).** The Ru/SiO_2_ in this study was prepared using a similar method as for the Ru/SBA-15 except the use of commercial SiO_2_ in place of the SBA-15.

**Synthesis of free Ru nanoparticles.** The free Ru NPs without support was prepared as follows: 1.06 mg RuCl_3_·2H_2_O was dissolved in 10 mL of distilled water, and then 20 mg of NaBH_4_ was added into the solution with vigorous shaking, and then used for the catalytic reactions.

**Synthesis of hydrazine borane (N_2_H_4_BH_3_, HB)**

Hydrazine borane was synthesized using the reported method.^S1^ Hydrazine hemisulfate salt (21.42 g) and sodium borohydride (10 g) were suspended in anhydrous dioxane (80 mL), and kept stirring for 48 h under Ar atmosphere. Afterwards, the mixture was separated by centrifugation at 12000 rpm for 12 min. Then, the solvent was removed under vacuum at 313 K overnight and *n-*pentene was added to precipitate HB. Finally, the precipitate was separated by centrifugation, washed with *n-*pentene for several times, and dried under vacuum at 313 K. The obtained material is a white solid with a purity of > 99% verified by ^1^H NMR and XRD. ^1^H NMR (*δ*/ppm, probe head Dual ^1^H/^13^C, 300.13 MHz, CD_3_CN, 30 ºC, *J*/Hz): 5.45 (s, 2H, B-NH_2_), 3.44 (s, 2H, N-NH_2_), 1.42(q, 3H, ^1^*J*_HB_=95 Hz).


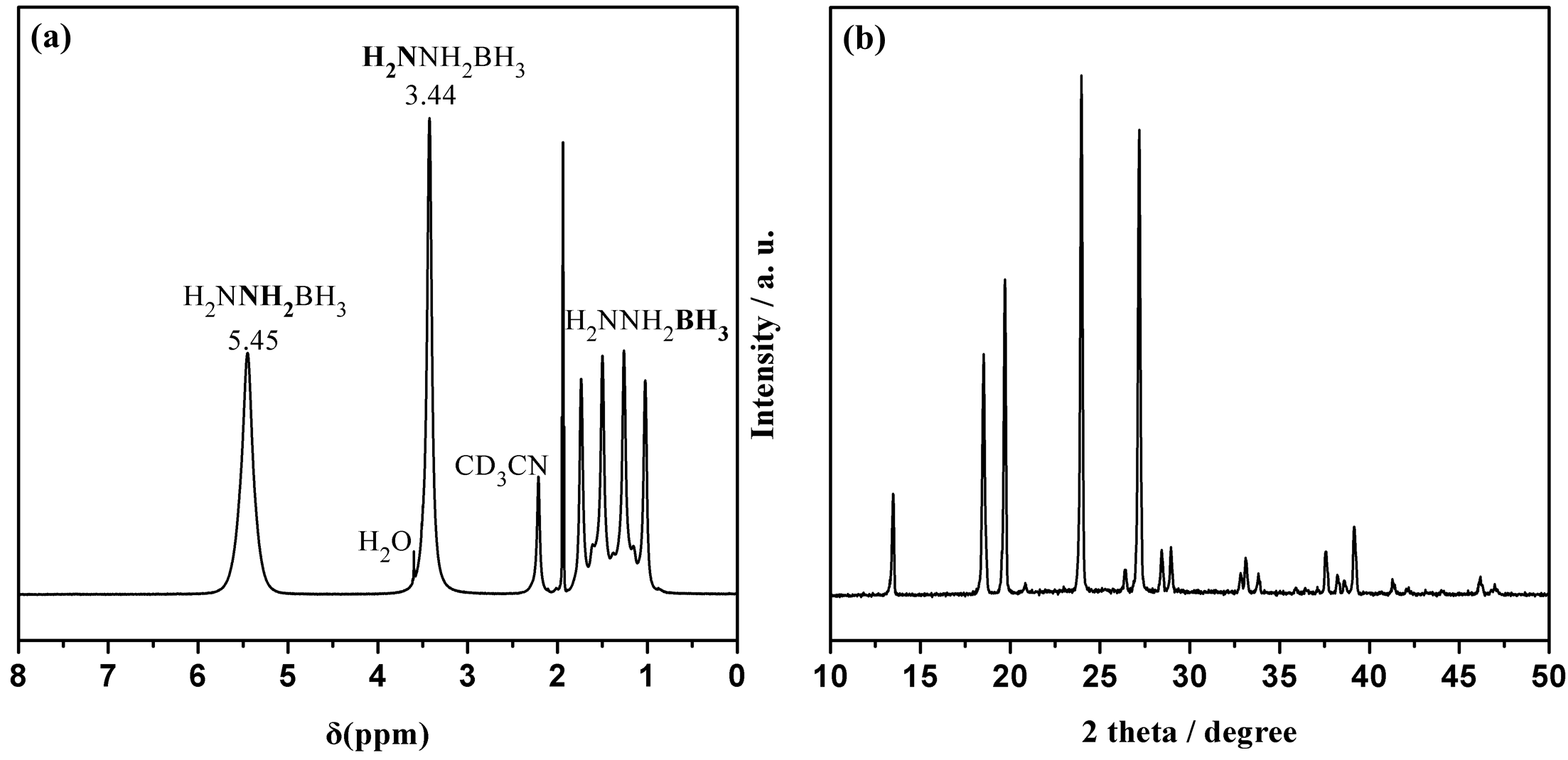


**Figure S1** |(a) Solid-state ^1^H NMR spectrum and (b) XRD pattern of the obtained HB.


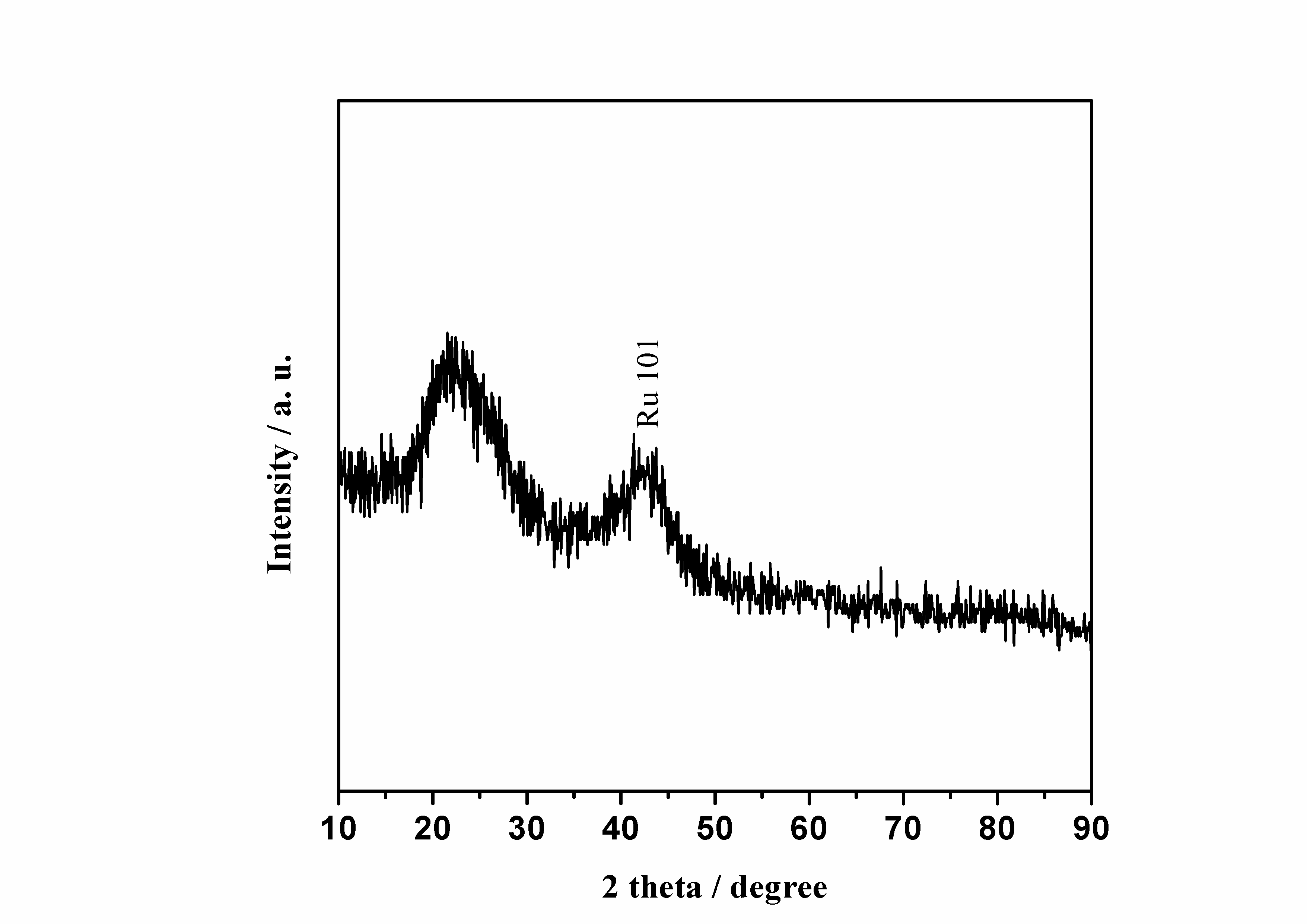


**Figure S2** |XRD patterns for the synthesized Ru@SBA-15 NCs with 20 wt% Ru loading.


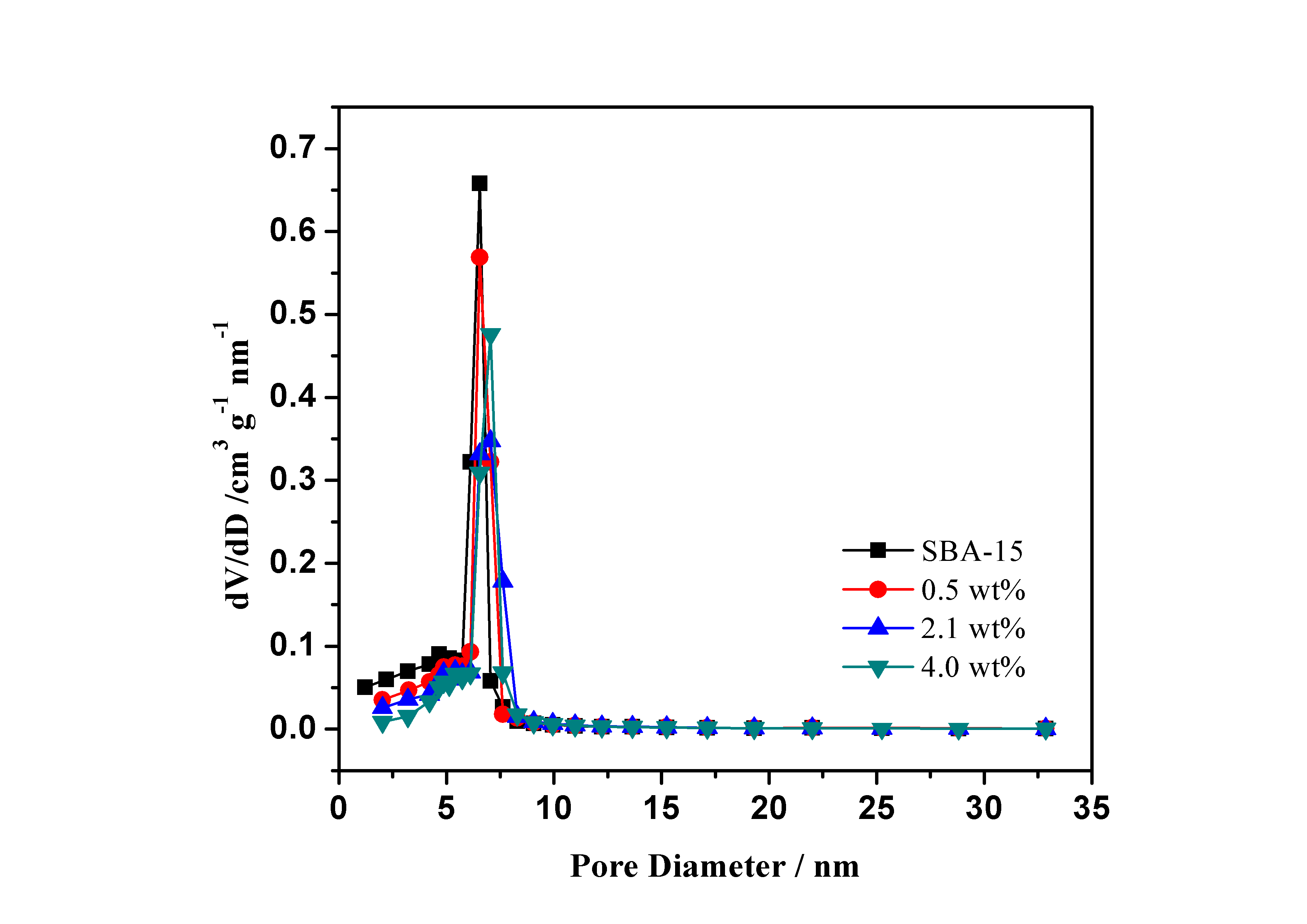


**Figure S3** | Pore size distribution of SBA-15 and Ru@SBA-15 NCs with different Ru loadings.


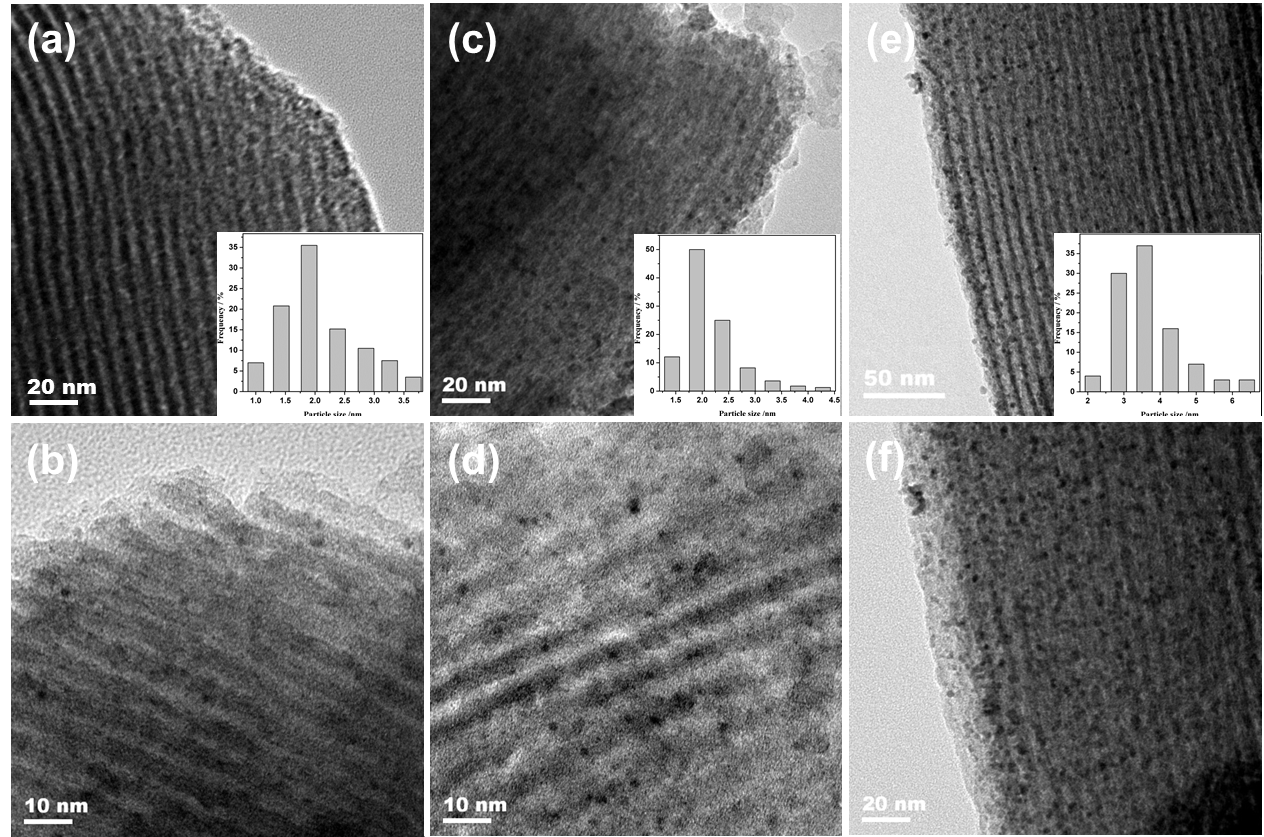


**Figure S4** | TEM images of (a,b) 0.5 wt%, (c,d) 1.1 wt%, and (e,f) 4.0 wt% Ru loading of Ru@SBA-15 NCs. Inset: the corresponding particle size distribution.


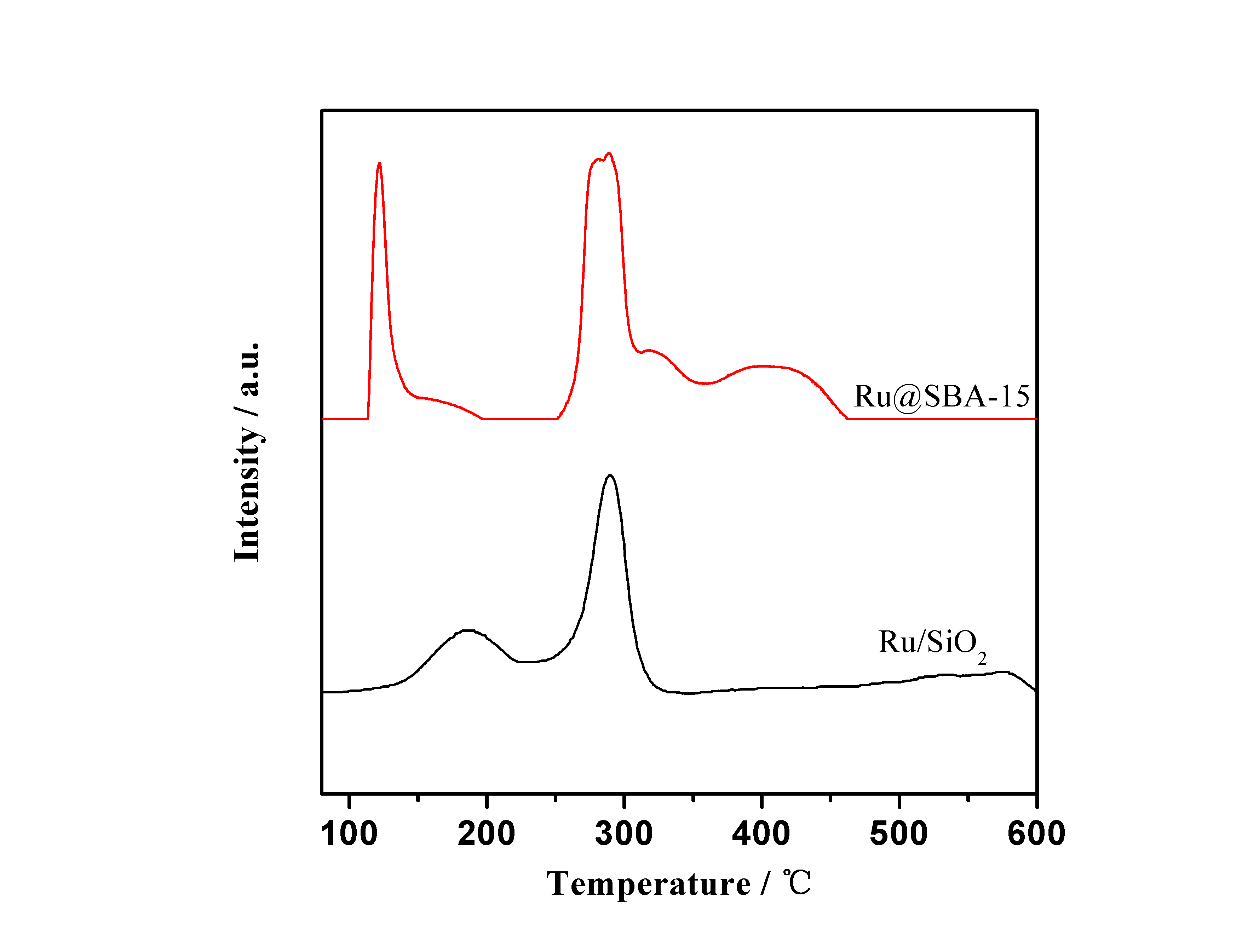


**Figure S5** | H_2_-TPR profiles for the Ru@SBA-15 and Ru/SiO_2_ NCs.

It can be found that the Ru@SBA-15 and Ru/SiO_2_ NCs both show two reduce peaks below 300 ^o^C, are at 117 ^o^C (185 ^o^C) and 286 ^o^C (290 ^o^C), respectively. The first low temperature peak can be ascribed to the reduction of well dispersed Ru oxides with small particle size, whereas the second peak can be assigned to Ru oxides with large particle size. In addition, there is a another higher temperature reduce peak (~420 ◦C) in the Ru@SBA-15 NCs compared to Ru/SiO_2_ NCs, indicating a much stronger interaction of Ru with SBA-15 support (Ru-O bonds) in Ru@SBA-15 NCs as confirmed by XPS spectrum (Fig. 5b).


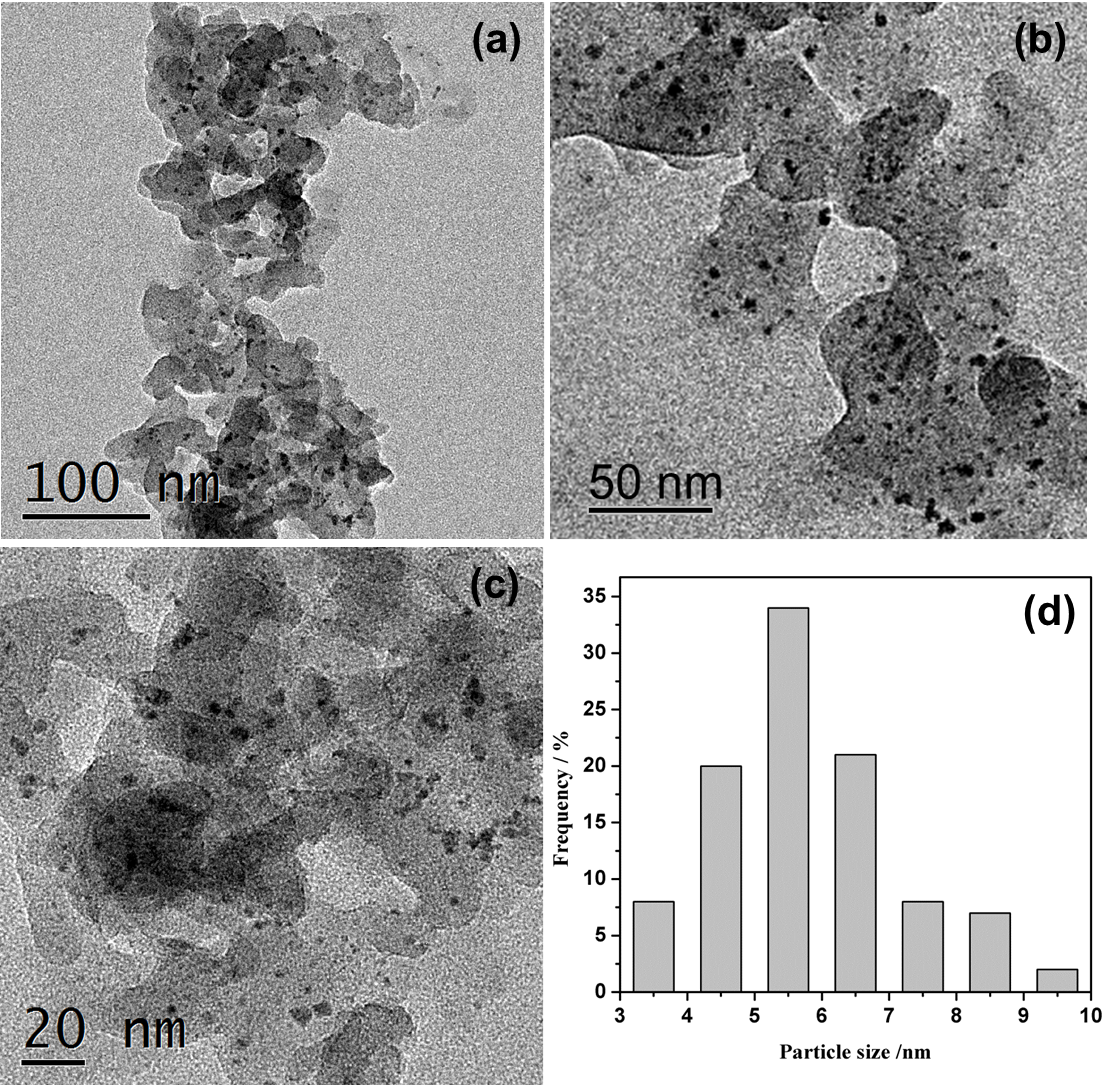


**Figure S6 |** (a-c) TEM images and (d) particle distribution of Ru/SO_2_ NCs with 2.1 wt% Ru loading.


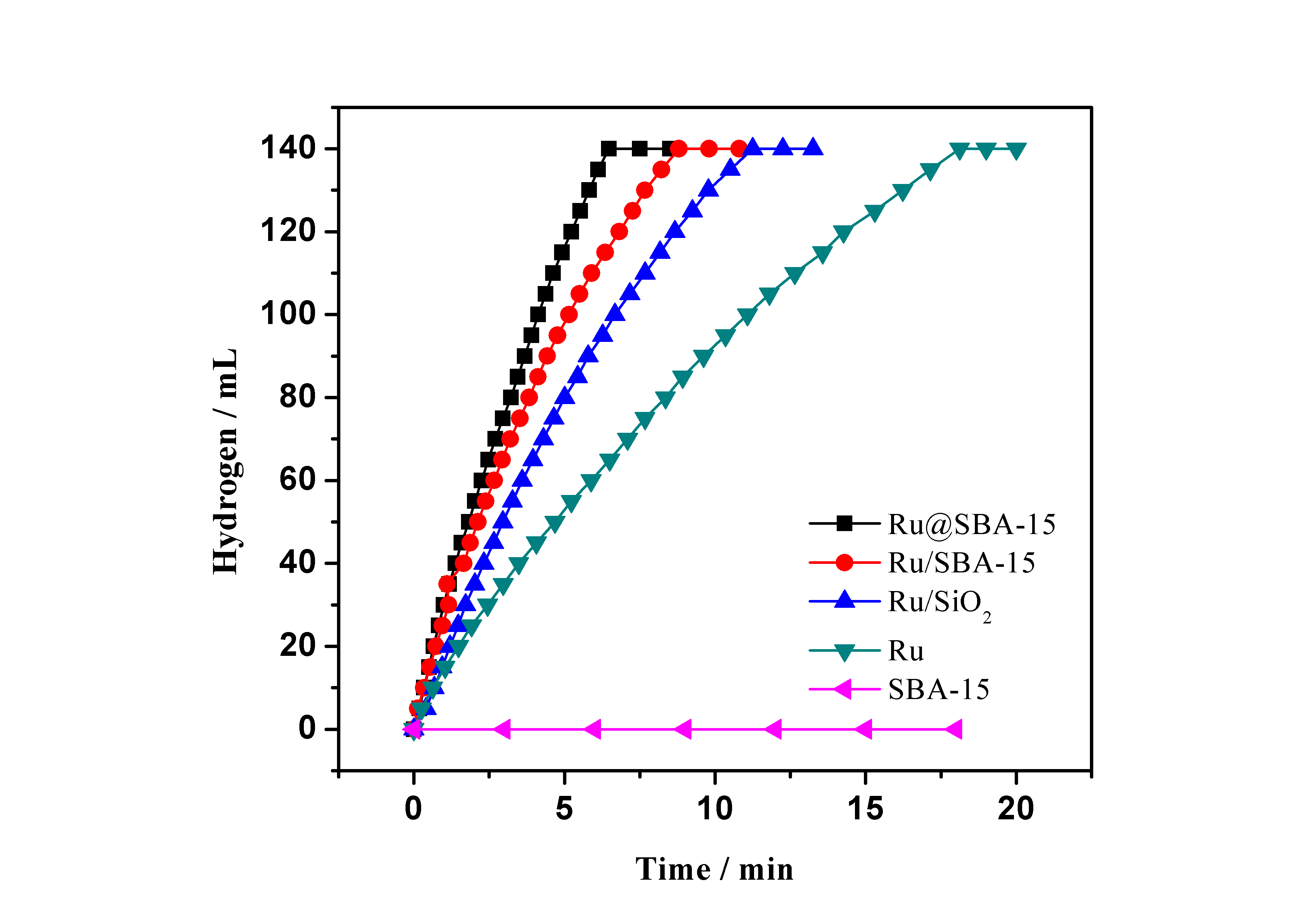


**Figure S7** | Hydrogen generation from the hydrolysis of AB (200 mM, 10 mL) catalyzed by Ru@SBA-15, Ru/SBA-15, Ru/SiO_2_, free Ru NPs, and SBA-15 at 298 K (Ru/AB=0.002).


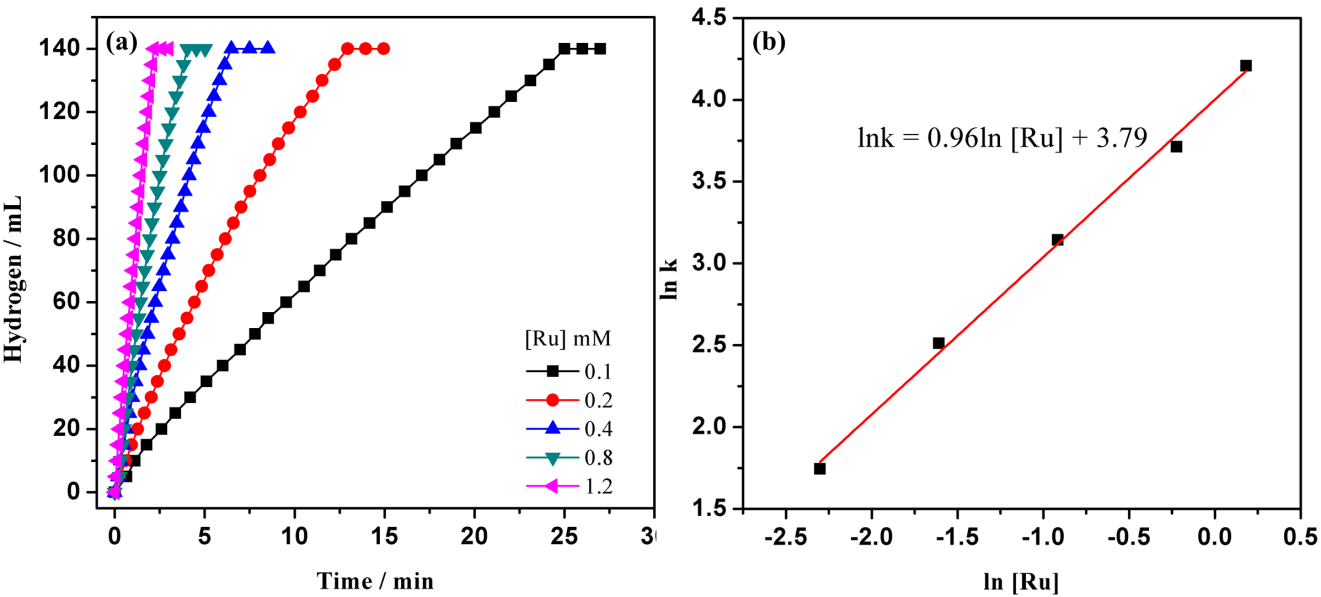


**Figure S8** | (a) Hydrogen generation from the hydrolysis of AB (200 mM, 10 mL) catalyzed by Ru@SBA-15 NCs with different catalyst concentration at 298 K. (b) Plot of the hydrogen generation rate versus ruthenium concentration, both in logarithmic scale.


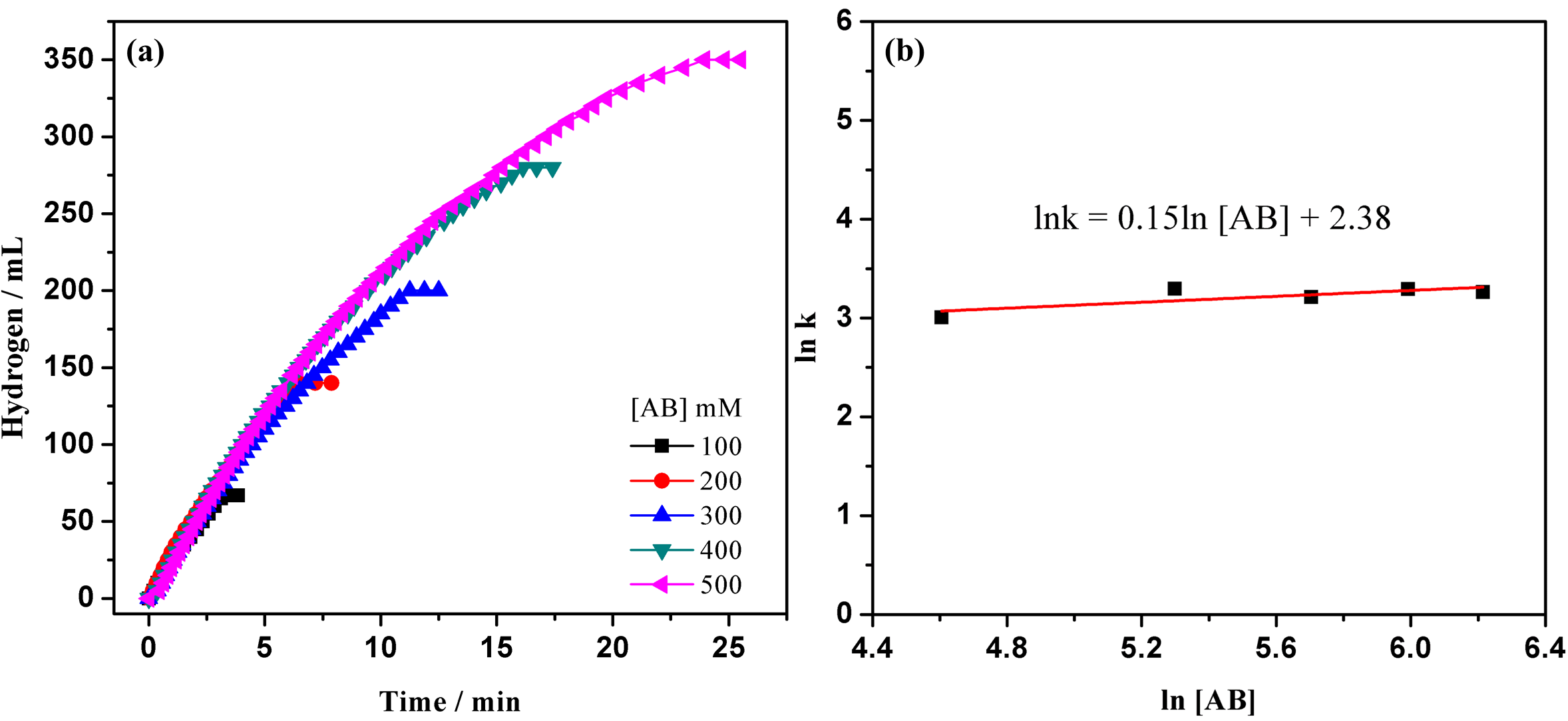


**Figure S9** | (a) Hydrogen generation from the hydrolysis of AB starting with different initial concentrations of AB while keeping the catalyst concentration constant at 0.4 mM Ru at 298 K. (b) Plot of the hydrogen generation rate versus AB concentration, both in logarithmic scale.


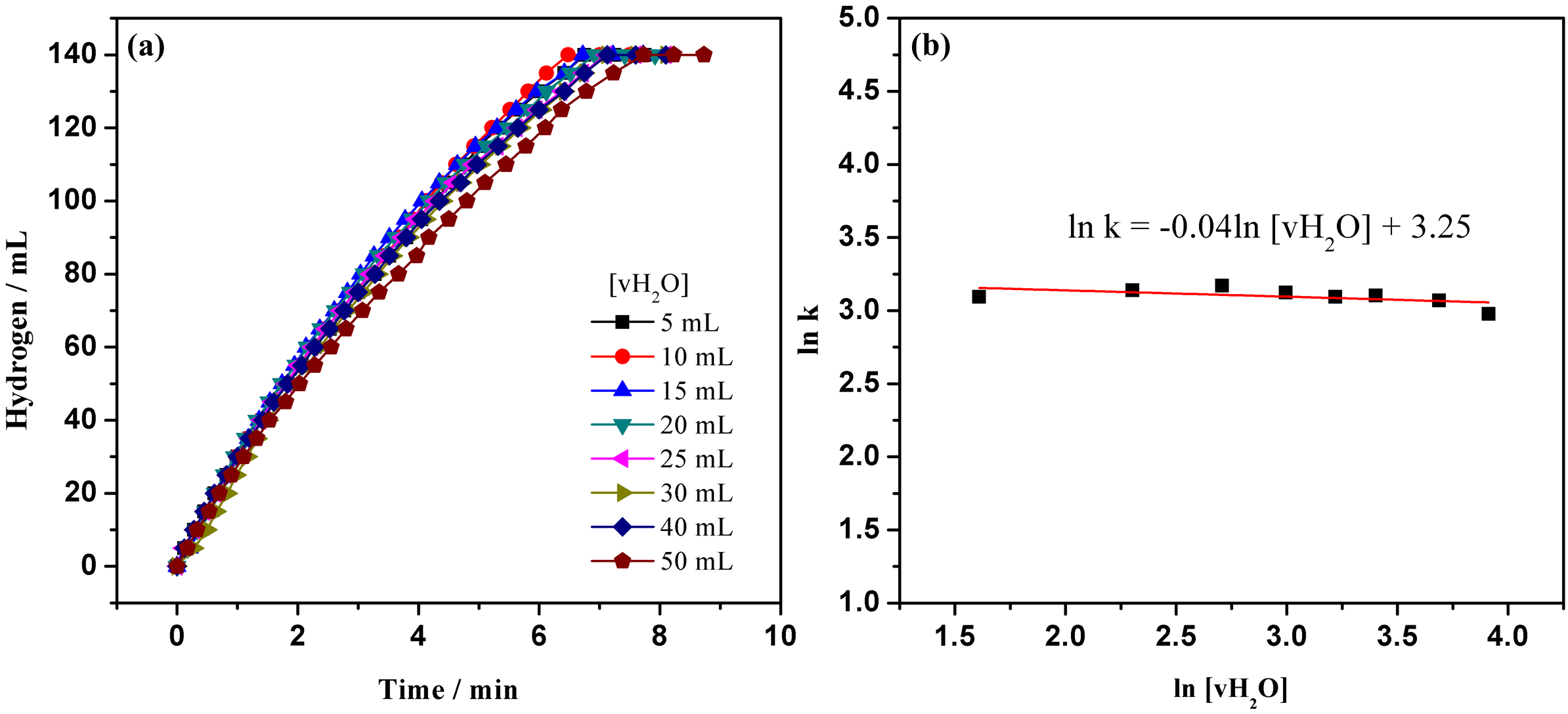


**Figure S10** | (a) Hydrogen generation from the hydrolysis of AB catalyzed by Ru@SBA-15 NCs with different H_2_O volumes at 298 K (Ru/AB = 0.002). (b) Plot of the hydrogen generation rate versus H_2_O volume, both in logarithmic scale.


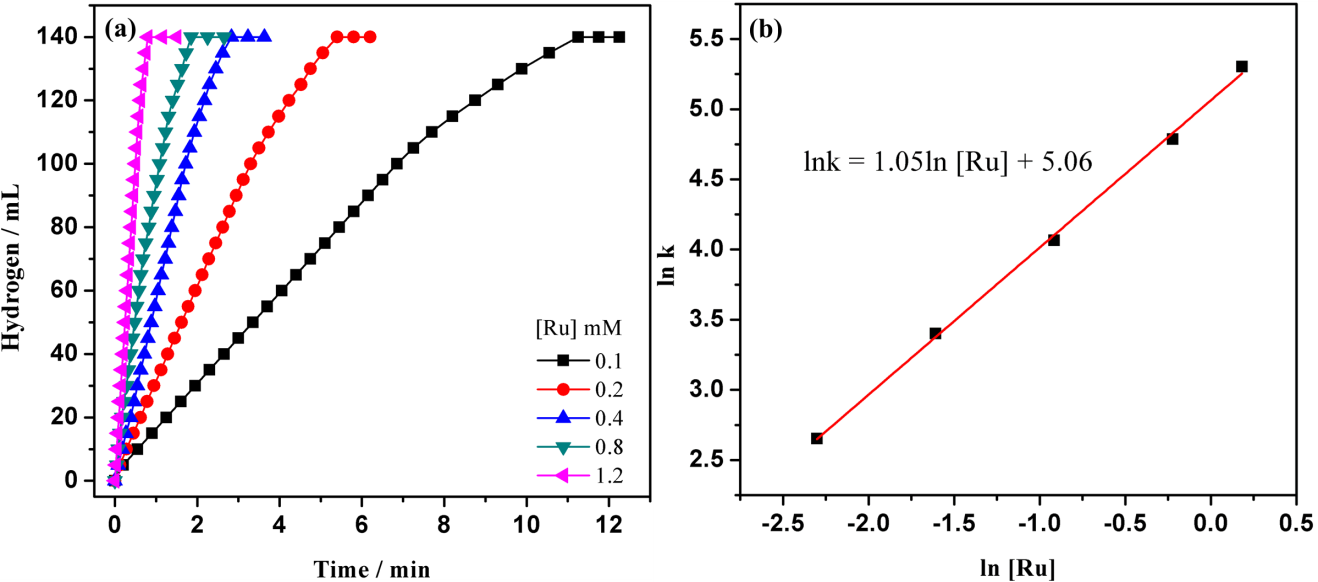


**Figure S11** | (a) Hydrogen generation from the hydrolysis of HB (200 mM, 10 mL) catalyzed by Ru@SBA-15 NCs with different catalyst concentration at 298 K. (b) Plot of the hydrogen generation rate versus ruthenium concentration, both in logarithmic scale.


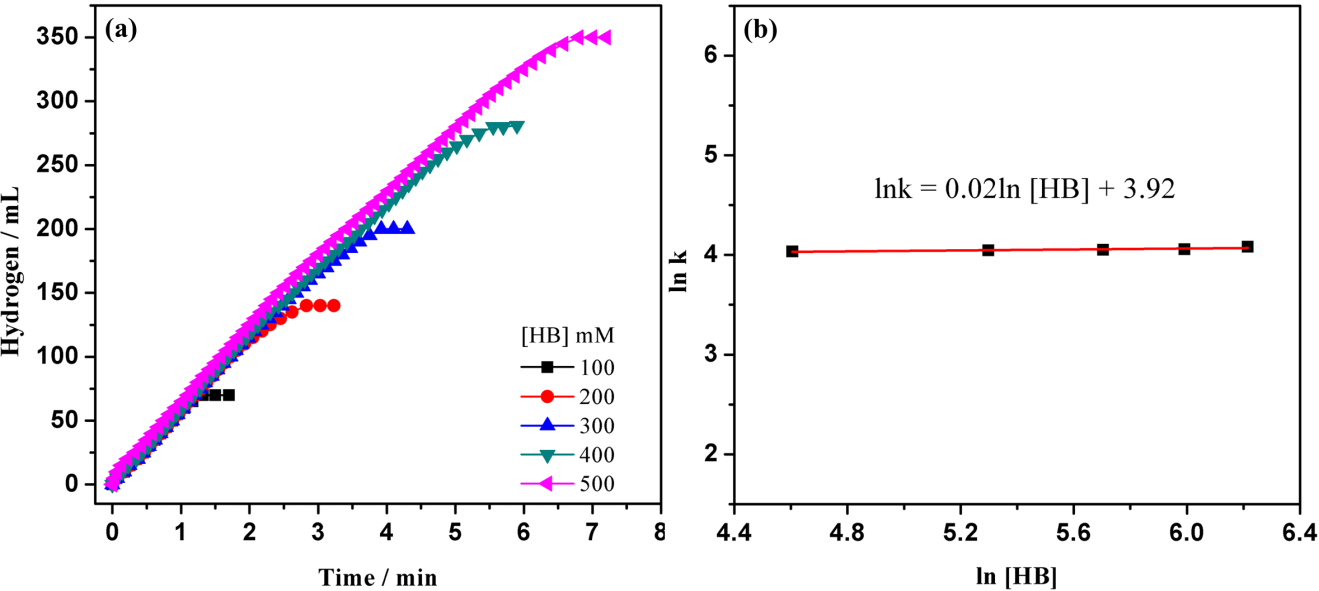


**Figure S12** | (a) Hydrogen generation from the hydrolysis of HB starting with different initial concentrations of HB while keeping the catalyst concentration constant at 0.4 mM Ru at 298 K. (b) Plot of the hydrogen generation rate versus HB concentration, both in logarithmic scale.


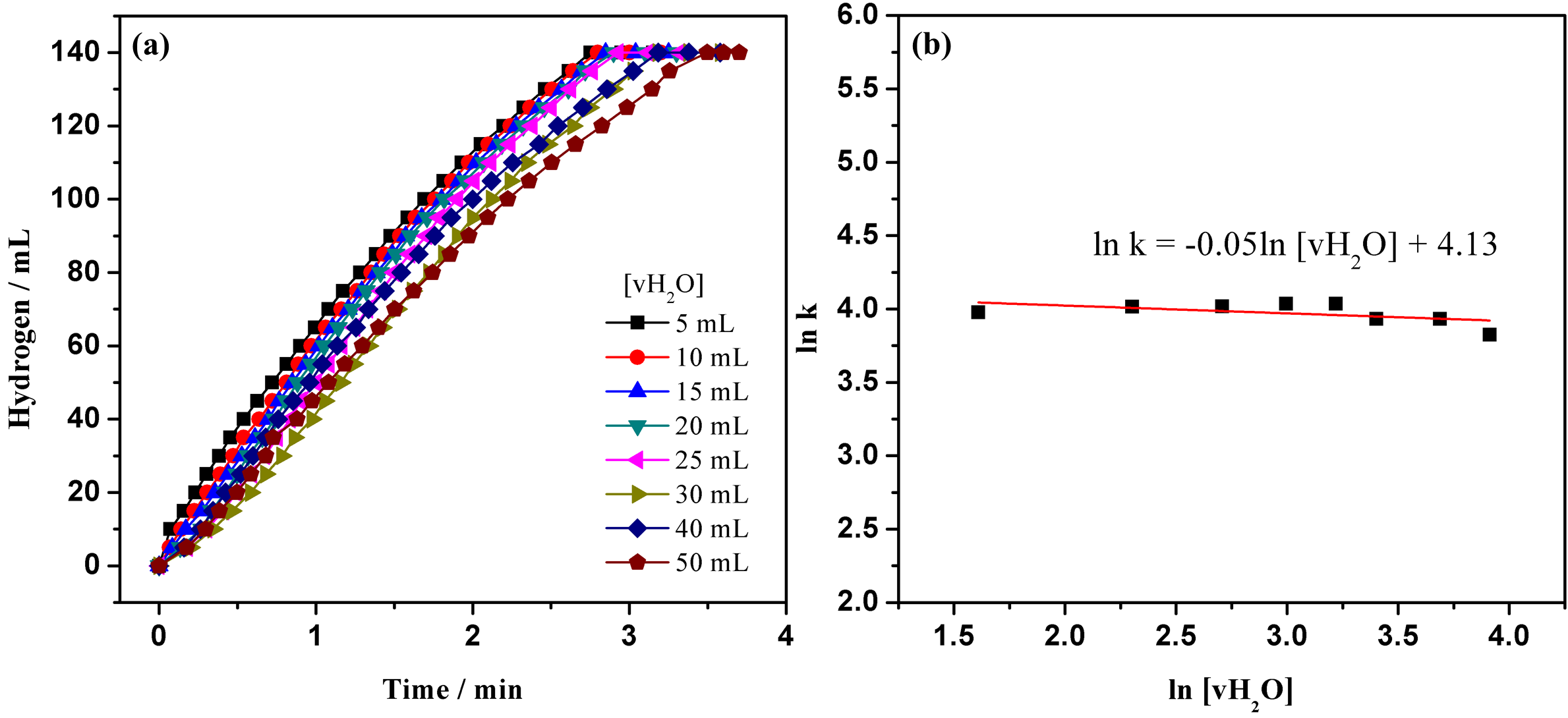


**Figure S13** | (a) Hydrogen generation from the hydrolysis of HB catalyzed by Ru@SBA-15 NCs with different H_2_O volumes at 298 K (Ru/HB = 0.002). (b) Plot of the hydrogen generation rate versus H_2_O volume, both in logarithmic scale.


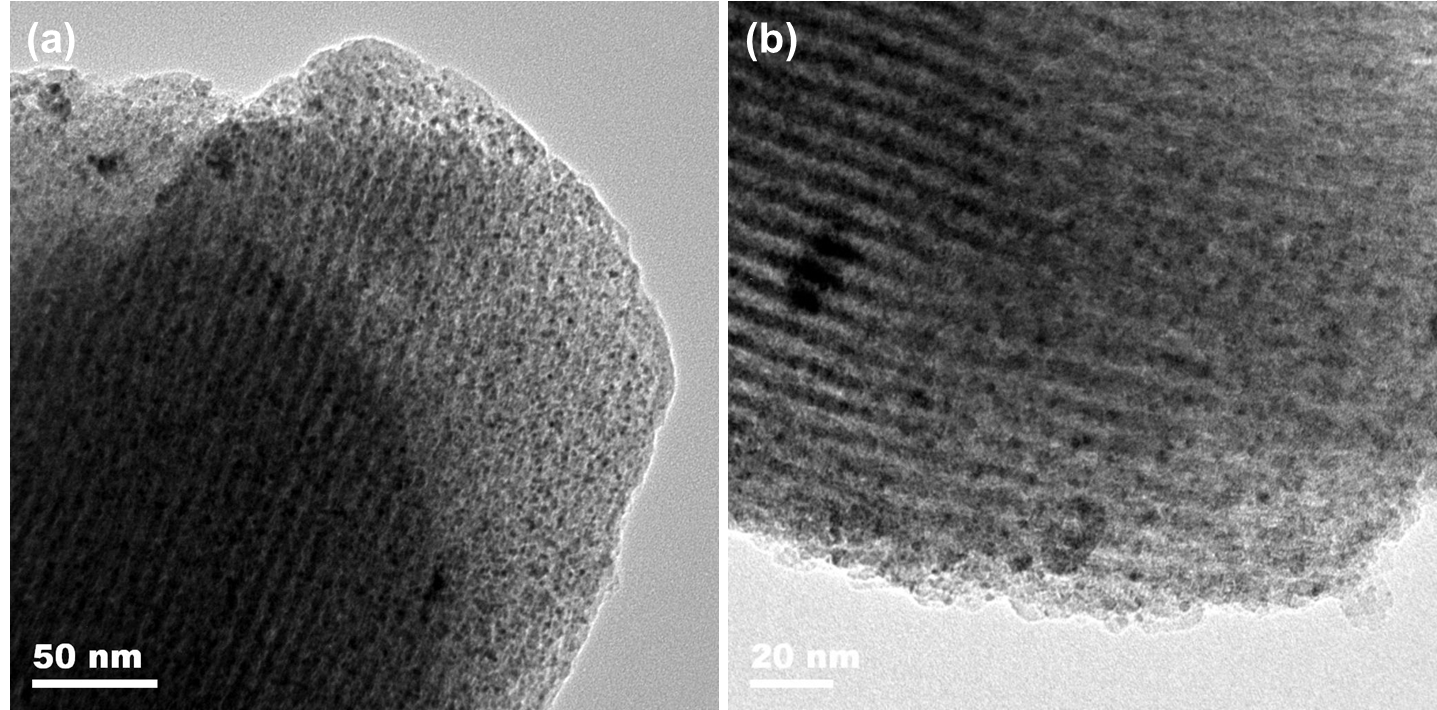


**Figure S14** | TEM images of Ru@SBA-15 NCs with 2.1 wt% Ru loading after recycle test.

**Table S1.** Catalytic activity of different Ru-based catalysts used for the hydrolysis of AB.

| Catalyst | T  (K) | *n*H_2_/*n*AB | *n*Ru/*n*AB | t  (min) | **TOF* (min^-1^) | *E*_a_  (kJ mol^-1^) | Ref. |
| --- | --- | --- | --- | --- | --- | --- | --- |
|  |  |  |  |  |  |  |  |
| Ru/g-C_3_N_4_ | 298 | 3 | 0.0013 | ~6 | 384.6 | 37.4 | 38 |
| Ru/CB | 298 | 3 | 0.0085 | ~1.08 | 326.8 | 34.81 | 41 |
| Ru@SBA-15 | 298 | 3 | 0.002 | 6.48 | 316 | 34.8 ± 2 | This work |
| Ru@SiO_2_ | 298 | 3 | 0.0025 | 6 | 200 | 38.2 | 35 |
| Metastable Ru | 298 | 3 | 0.0025 | ~6 | 200 | 27.5 | 52 |
| PSSA-co-MA stabilized Ru | 298 | 3 | 0.007 | ~2.28 | 187.6 | 54 | 53 |
| Ru@MWCNT | 298 | 3 | 0.00189 | ~8.9 | 178.3 | 33±2 | 34 |
| Ru(0)/SiO_2_-CoFe_2_O_4_ | 298 | 3 | 0.00097 | ~20 | 159.6 | 45.6 | S2 |
| Ru@TiO_2_ | 298 | 3 | 0.0012 | ~20 | 125 | 70 ± 2 | 43 |
| Ru@MIL-101 (1.62 wt%) | 298 | 3 | 0.008 | ~3.33 | 112.6 | 51.12 | 37 |
| Ru@ZK-4 | 298 | 3 | 0.005 | ~6.65 | 90.2 | 28.2 | 40 |
| Ru/graphene | 298 | 3 | 0.01 | 3.5 | 85.7 | 11.7 | S3 |
| RuNPs@nano-HAp | 298 | 3 | 0.0025 | 15 | 80 | 55 ± 2 | 54 |
| Ru(0)@X-NW | 298 | 3 | 0.00271 | ~13.9 | 79.6 | 77 ± 2 | S4 |
| Laurate-stabilized Ru | 298 | 3 | 0.005 | 8 | 75 | 47 | 55 |
| Ru/Hydroxyapatite | 298 | 3 | 0.00784 | ~ 6 | 64.1 | 58 ± 2 | S5 |
| Ru/γ-Al_2_O_3_ | 298 | 3 | 0.018 | 3 | 55.5 | 23 | 42 |
| Ru@Al_2_O_3_ | 298 | 3 | 0.005 | ~30 | 20 | 48 ± 2 | 56 |

***Calculation method for *TOF*:**

The turnover frequency (*TOF*) reported in this work was calculated based on the surface Ru atoms in catalyst, which is calculated from the equation as follow (eqn S1):

$TOF=\frac{\text{n}_{\text{H}_{2}}}{\text{n}_{\text{Ru}}\times D_{Ru}\times\text{t }}$ (S1)

Where *n_H2_* is the mole number of generated H_2_, *n_Ru_* is the mole number of Ru in catalyst, D_Ru_ is the metal dispersion of Ru, and *t* is the completed reaction time in minute. The metal dispersion of Ru (D_Ru_) on the SBA-15 was determined by H_2_ chemisorption on a Micromeritics AutoChem II 2920 automated catalyst characterization system. The D_Ru_ was then estimated by assuming the adsorption stoichiometry of one H atom per Ru surface atom (eqn S2). The D_Ru_ of Ru@SBA-15 was calculated to be about 73%.

$D_{Ru}=\frac{2\times\text{n}_{\text{H}_{\text{2 }}\text{chemisorbed on the Ru surface}}}{\text{n}_{\text{total Ru }}\text{ }}\times100$ (S2)

**Table S2.** Catalytic activity of different catalysts used for the hydrolysis of HB.

| Catalyst | *TOF*  (min^-1^) | *E*_a_  (kJ mol^-1^) | Ref. |
| --- | --- | --- | --- |
| Ru@SBA-15 | 726 | 41.3 ± 2 | This work |
| RhCl_3_ | 200 | 44 ± 2 | 14 |
| Rh@HAP | 111.7 | 45 | 15 |
| Cu@SiO_2_ | 7.58 | 51 ± 2 | 30 |
| PSSMA-Co | 6.2 | 60 ± 2 | S6 |
| NiCl_2_ | 3.05 | 73 ± 2 | 16 |
|  |  |  |  |

**References**

1. Gunderloy, F. C. Hydrazine-mono- and bisborane. *Inorg. Synth.* **9**, 13-16 (1967).
2. Akbayrak, S. Kaya, M. Volkan, M. & Özkar, S. Ruthenium(0) nanoparticles supported on magnetic silica coated cobalt ferrite: Reusable catalyst in hydrogen generation from the hydrolysis of ammonia-borane. *J. Mol. Catal. A: Chem*. **394**, 253-261 (2014).
3. Cao, N. Luo, W. & Cheng, G. Z. One-step synthesis of graphene supported Ru nanoparticles as efficient catalysts for hydrolytic dehydrogenation of ammonia borane. *Int. J. Hydrogen Energy* **38**, 11964-11972 (2013).
4. Akbayrak S. & Özkar, S. Ruthenium(0) nanoparticles supported on xonotlite nanowire a long-lived catalyst for hydrolytic dehydrogenation of ammonia-borane. *Dalton. Trans*. **43**, 1797-1805 (2014).
5. Akbayrak, S. Erdek, P. & Özkar, S. Hydroxyapatite supported ruthenium(0) nanoparticles catalyst in hydrolytic dehydrogenation of ammonia borane: Insight to the nanoparticles formation and hydrogen evolution kinetics. *Appl. Catal. B: Environ*. **142-143**, 187-195 (2013).
6. Karahan, S. & Özkar, S. Poly(4-styrenesulfonic acid-co-maleic acid) stabilized cobalt(0) nanoparticles: A cost-effective and magnetically recoverable catalyst in hydrogen generation from the hydrolysis of hydrazine borane. *Int. J. Hydrogen Energy* **40**, 2255-2265 (2015).
